# Supplementary material for: Are carbon emissions trading and green financial instruments synergistic? -Comprehensive quantitative research based on content analysis
Source: PLoS One. 2024 Mar 7;19(3):e0298601. doi: 10.1371/journal.pone.0298601 (PMC10919601; doi:10.1371/journal.pone.0298601)
Supplement: S1 Data — (ZIP) [file pone.0298601.s001.zip › Data/地级市Z_F工作报告.zip!/║■─╧/│ñ╔│╩╨/└┤╫╘╣1⁄2╓┌║┼í╛┬φ┐╦╩2╛▌═°í┐_2009.docx]

各位代表：

现在，我代表市人民政府向大会作工作报告，请予审议，并请各位政协委员和其他列席人员提出意见。

一、2008年工作回顾

2008年是新一届政府履职的第一年。一年来，面对一系列重大事件和严峻考验，在市委的坚强领导下，我们坚持解放思想、科学发展，围绕建设创业之都、宜居城市、幸福家园，率先基本实现全面小康的目标，抓住“两型社会”建设综合配套改革试验的重大机遇，迎难而上，团结拼搏，圆满完成了市第十三届人民代表大会第一次会议确定的各项目标任务。

——经济平稳快速增长。地区生产总值增长15%以上（预计数，下同）；工业总产值达3100亿元，跃居全国省会城市第10位；地方财政收入达340亿元，增长29.5%；完成全社会固定资产投资1850亿元，增长28%；社会消费品零售总额1270亿元，增长22.5%。

——城市形象明显提升。重点整治“七路二桥”28个堵点，城区通行能力提高20%以上，道路更加通畅；全面整治扬尘和废气污染，空气质量优良率达90%，空气更加清新；拆除违章户外广告和门店招牌220万平方米，完成363个社区标准化建设，市容更加整洁，被评为国家园林城市。

——农村经济加速发展。粮食总产量达281.1万吨，粮食单产、总产创近五年新高；完成农产品加工销售收入360亿元，增长23.5%；宁乡县继长沙县、浏阳市之后进入全国县域经济基本竞争力与科学发展百强县（市）。

——先导区建设全面启动。设立长沙大河西先导区并正式挂牌运作；完成大河西空间发展战略规划，实施重点建设项目“三年行动计划”，开工建设重大项目52个；全面启动梅溪湖、洋湖垸、岳麓山景区和滨江新城4大片区开发工作。

——开放领域日益拓展。被确定为全国服务外包基地城市，高新区成为示范区；新引进银行和保险机构4家；新增长沙至大阪国际航线，开通长沙至深圳“五定班列”；与美国安纳波利斯市结为友好城市。全年实际到位外资18.02亿美元，进出口总额51.5亿美元，分别增长19.8%、26.5%。

——人民生活持续改善。全年新增城镇就业10.2万人，新增农村转移就业8.11万人，城镇登记失业率3.6%；居民消费价格指数105.4，居全国省会城市倒数第7位；城乡居民人均可支配收入分别达18253元和7353元，增长13%和16%。

——社会保障成效明显。社会保险覆盖面不断扩大，城镇居民基本医疗保障制度顺利推进，实施被征地农民社会保障制度；城乡居民低保补助月均提标50元和20元，实现应保尽保；发放经济适用房货币补贴凭证1.1万户，建设(筹集)廉租房48.7万平方米；城乡义务教育实现“一费制”全免；“新农合”参合率达94.1%。

——政府管理不断创新。利用各种方式广泛听取群众意见，实行市民代表列席政府常务会议和市长办公会议制度；聘请市民作为专项工作顾问；推行审批流程再造，行政审批时限压缩30%以上；全面实行土地出让网上招拍挂；率先全国开展环境资源补偿交易；实行政府采购与监管分离制度；建立市区统一的公共资源交易平台。

各位代表，这些成绩的取得来之不易。一年来，我们经受的挑战空前严峻，取得的成效超过预期。面对历史罕见的冰冻灾害，主动把握、科学决策，奋力组织抗灾，开展生产补损，把冰灾损失降到最低程度，经济运行迅速步入正轨；面对资源性供电紧张，实行调煤保电、有序供电和节约用电，有效地稳定了生产、稳定了市场、稳定了民心；面对房产市场需求不足，启动住房制度改革，推行经济适用房货币补贴，保持了房产市场平稳发展；面对金融危机的严峻考验，抢抓宏观调控机遇，开展服务经济大行动，加大项目建设力度，出台扶持企业发展政策，为经济发展注入了动力和活力。在这一系列困难和挑战面前，我们见事早、判断准、行动快，做到了“早半拍”决策，赢得了时间，抢到了先机，经受了考验，增强了攻坚克难的信心和勇气！

在加快发展中，我们着力推进了以下几个方面的工作：

（一）加速推进新型工业化。突出工业重大项目建设，开工建设亿元以上重点项目136个。突出骨干企业和产业集群发展，形成年销售收入过300亿元企业1家、过200亿元企业2家；六大优势产业集群总产值占规模工业总产值的42%，工程机械产业集群产值突破500亿元，生物医药、新材料产业集群快速发展。突出高新技术产业和园区工业发展，高新技术产业产值达1120亿元，增长22.2%；“两区九园”工业总产值达1470亿元，增长40%；工业对经济增长的贡献率达50%以上。工业成为经济增长的首要支撑。

（二）着力建设现代宜居城市。坚持高起点规划，完善城乡规划编制体系，编制并落实城市景观、建筑色彩等11个专项规划；严格执行规划，对跃进湖、西湖还湖于民。坚持精心建设，树立全寿命周期成本理念，以“一江两岸”和片区开发建设为重点，城建重点工程投资136亿元，完成长沙大道、年嘉湖隧道、湘江防洪道路等市政工程，建成金洲大道、橘子洲风景区等精品工程。坚持精细管理，以创建全国文明城市为契机，突出治脏、治乱、治差，抓好小区、小巷、小院、小店改造，实施楼栋美化、亮化和绿化提质工程，改造棚户区60万平方米，加强供电、供水、供气和公共客运保障，提升窗口行业文明服务水平。虽然我们与全国文明城市的荣誉擦肩而过，但全市人民付出了巨大的努力，市容状况、卫生状况、交通状况明显改观，文明程度大幅提升。长沙的知名度和影响力不断扩大。

（三）深入推进新农村建设。坚持把解决“三农”问题作为全部工作的重中之重。加强村镇规划，完成村规划216个，覆盖率达53%以上；实现建制镇规划全覆盖。加强农村基础设施建设，新建改造农村公路2030公里；新解决13.42万人饮水安全问题；新建沼气池1.5万个；完成农村电网改造投资3.9亿元；加快“万村千乡”市场工程建设。推进四大农业优势产业带建设；发展农民专业合作组织460家。全面实行重大动物疫病免费强制免疫，全年无重大动物疫病发生。推进新农村示范村建设和农村环保“三新”行动，新造生态林10万亩，环保村民自治模式得到国务院肯定。农村面貌发生新的变化。

（四）强势启动“两型社会”建设。坚持生态优先，开展绿色城乡建设标准体系研究，新建建筑全部执行节能标准，新增项目实行能耗审核和节能评估制度，实施循环经济示范工程，单位地区生产总值综合能耗下降4%，主要污染物排放总量减少4%。启动环境保护三年行动计划，加大污水、垃圾处理等设施建设。编制先导区规划和重点地区控规，加快坪塘老工业基地产业退出，改革行政审批、土地管理和投融资机制。“两型社会”建设成为引领长沙发展的新航标。

（五）积极发展现代服务业。突出发挥服务业的既有优势。大力发展生产性服务业，成功举办和组织欧美招商会、海外华商潇湘行、全国知名民营企业家长沙行等活动，引进一批重大项目。发展现代物流业，金霞保税物流中心通过国家审批。成功举办和承办全国糖酒交易会、生物产业大会、科技成果转化交易会、现代农业投资合作会、长沙车展等重大会展活动，荣获“全国十佳节庆城市”称号。加快发展金融、文化创意、电子商务等服务业，新增上市公司2家。继续抓好商贸流通、旅游、餐饮等服务业发展，入境旅游收入居中部城市首位。加强对外经贸合作，帮助企业拓展国际市场。服务业继续发挥对经济增长的支撑作用。

（六）加快社会事业发展。加强产学研金合作，加快科技成果转化和产业化。荣获国家知识产权示范城市称号。推进教育强市建设，促进各类教育均衡发展。发展公共文化，切实保护非物质文化遗产，市属文博单位全部免费开放。完成乡镇（村）卫生院（室）改造，基本构建城区“15分钟医疗服务圈”。广泛开展全民健身运动，顺利完成奥运会和残奥会火炬传递。完成第七届村委会换届工作。发挥广播电视、新闻出版的舆论导向作用。认真落实党的民族、宗教政策。人口自然增长率为4.95％。，连续七年被评为省计划生育模范市。统计、台办、市志、档案等事业取得新的进步。

（七）努力构建和谐长沙。加快以改善民生为重点的社会建设。市本级民生保障支出占一般预算支出的79%，提高3个百分点。共投入78.5亿元，为民办实事工程任务全面完成。完成50个农贸市场提质建设。加强社会救助、优抚、慈善等工作，落实扶持困难群体价格优惠政策。深化平安长沙建设，开展领导干部大接访活动。加强国家安全、国防建设和人防工作，驻长部队在抗击冰灾等突发事件中发挥了中流砥柱作用。启动食品安全城市建设，及时处置“问题奶粉”事件。气象、水文、地震、公积金管理、海事、移民开发等部门以及工会、共青团、妇联、科协、文联、侨联、红十字会、关工委、老龄委等群团组织为长沙发展做出新贡献。

（八）全面加强政府建设。建设服务政府、效能政府、阳光政府是对全市人民的庄严承诺。坚持民主决策，倾听群众意见，对城市农贸市场改建、出租车价格调整等重大事项进行公示、听证。坚持科学决策，尊重专家意见，通过优化设计，一期疏导工程计划投资3.93亿元实际投资仅1.67亿元， 6条主干道实施改造节约土地29.94公顷。坚持依法行政，自觉接受人大及其常委会的法律监督、工作监督和政协的民主监督，认真办理人大代表建议、批评、意见和政协委员提案，开展“五五”普法教育。坚持严格管理，强化预算约束，加强审计监督，完成政府投资审计项目340个；启动行政许可网上审批，在线审批项目达30%；加强国资监管，推进企业社会职能分离和政策性破产工作；加强目标管理，逐项分解落实政府工作报告的任务。坚持廉洁从政，加强公务员队伍建设，强化行政监察，抓好治理商业贿赂和行风评议，严肃查处各类案件。政府与人民群众的联系更加密切，政府形象不断提升。

各位代表，上述工作凝聚了全市人民的智慧和汗水，是全市上下团结奋斗的结果。在此，我谨代表市人民政府，向全市各族人民，向驻长部队官兵，向公安政法干警，向各民主党派、工商联、人民团体和各界人士，向所有关心支持长沙发展的朋友们，表示衷心的感谢！

回顾一年来的工作，我们深刻体会到：必须坚持解放思想，创新理念。用科学发展观指导思想大解放，以国内领先和国际先进水平为坐标，提出工作要求，确定发展目标，以新的理念推进产业发展和城乡建设，用新的思路和方法推动政府工作。实践证明，只有继续解放思想、改革创新，才能找到破解难题的有效办法。必须坚持以人为本，民生为先。民之所需，我之所应。始终把群众冷暖放在心上，把改善民生、服务群众放在首位，想问题、做决策、安排资金首先考虑群众受益。实践证明，只有真心实意地为群众办事，才能赢得群众的理解和支持。必须坚持民主决策，科学决策。多一份民主，多一份智慧。面对复杂的经济社会问题，注重调查研究，广泛听取意见，坚持集体决策，问计于民、问计于专家，增强决策的群众基础，提高决策的科学性。实践证明，只有发扬民主、广纳群言，我们的决策才能经得起实践和历史的检验。必须坚持规范管理，改进政风。坚持制度管事，管事务必从严。立足长远，不急功近利，不追求政绩工程，不搞形象工程。将人民群众的评价与监督作为改进政风的动力。实践证明，只有切实加强政府建设，清正廉明，才能不断提高政府的执行力和公信力，努力建设人民信赖的政府。

2008年尽管取得了新的成绩，但我们清醒地认识到，前进中还存在不少困难和不足：国际国内宏观经济形势对经济发展产生较大的不利影响，房地产业投资增幅下降对财政增收的影响明显，发掘替代财源还缺乏更多途径；城市规划执行不严格和建设管理方式比较粗放的现象依然存在；民生保障仍处于较低水平，城乡居民增收难度加大；安全监管存在薄弱环节，安全生产事故在个别领域时有发生；政府管理和政风建设需进一步加强，服务质量和办事效率与群众的期望还有差距，等等。对此，我们深感责任重大，必须采取有效措施认真加以解决。

二、2009年面临的形势与任务

各位代表，2009年将是新世纪以来经济发展面临困难最大、挑战最严峻的一年。金融危机的影响正逐步从金融领域向实体经济扩散、从沿海向内地扩散，对经济发展的影响进一步加深，经济运行困难增加，经济下行压力加大，企业经营困难增多，农民持续增收难度加大。对长沙的影响，总体判断是全面的、深刻的。主要表现为：企业运行艰难。企业订单减少，销售下降，开工不足，停产歇业增多，特别是中小企业面临更大困难。房产市场低迷。商品房销售量同比明显下降，空置房积压，加上大量在建房，房产市场供大于求，房地产业投资意愿下降。财政增收乏力。投资增幅下降，企业生产不景气，导致税收减少；国家实施一系列惠民措施，刚性支出增加，财政收支平衡面临严峻考验。就业压力加大。外出务工农民返乡增多，新增就业、下岗失业等因素叠加在一起，对就业形成巨大压力。潜在矛盾可能增多。失业人员增多，居民增收困难，生活压力加大，以及可能出现的企业“三角债”等因素，导致潜在的社会不稳定因素增加。

面对这些挑战，我们必须把形势估计得严峻一些，把困难考虑得充分一些，在思想上和工作部署上做好应对准备。同时，也要认识到，动力与压力同在，机遇与挑战并存，金融危机对不同主体、不同地区的影响是不一样的，我们面临的挑战前所未有，面临的机遇也前所未有，长沙具有诸多发展优势和有利条件：

一是具有国家宏观政策机遇。国家实施积极的财政政策、适度宽松的货币政策、扩大规模与优化结构并举的投资政策、更加优惠的税收政策、更加普惠的民生政策等，使我们一些多年想干而干不了的事可以干了，棚户区改造、地铁工程、过江隧道、污水管网建设等项目可以得以实施，形成强大的投资和需求拉动。通过企业投入不足政府投资补、产业投入不足基础设施投资补，将为长沙发展提供强有力的支撑。我们一定要抓住这个千载难逢的机遇。

二是具有良好的发展平台。“两型社会”建设为长沙提供了重大的发展机遇，打造了新的巨大平台，吸引和凝聚了人气。特别是先导区建设的全面启动，众多投资者前来长沙创业发展，一批重大项目先后落户先导区。随着先导区基础设施、生态工程、片区开发建设的快速推进，将吸纳更多的投资和项目落地，有效拉动经济增长。

三是具有经济结构性优势。长沙产业分布广，结构呈现多样性，内生动力充沛，国家扩大内需，将进一步促进我市工程机械等优势支柱产业发展；外贸依存度较低，在经济全球化背景下是一种劣势，在金融危机背景下劣势转化为优势，受到的直接冲击小；即使是受影响较大的房地产业，2008年在中部省会城市中销售面积居首位，在同类地区属最平稳的；服务业比较发达，一直是长沙的优势，特别是消费持续旺盛，社会消费品零售总额增速多年位居全国省会城市前列。经济的结构性优势使长沙抗击风险的能力增强，金融危机的影响相对其他城市可能要小。

四是具有坚实的发展基础。经过改革开放30年的持续快速发展，我们积累了雄厚的物质基础和综合实力。更为重要的是，我们有市委的坚强领导，驾驭复杂局势和领导发展的能力不断增强，全市干部群众凝聚了不畏困难、勇于担当、团结拼搏的精神力量，完全有能力应对和战胜任何困难与挑战。

基于上述分析，今年政府工作的总体要求是：以邓小平理论和“三个代表”重要思想为指导，深入贯彻科学发展观，按照“两型社会”建设和率先基本实现全面小康的要求，牢牢扭住经济建设中心，紧紧围绕群众最关心、最直接、最迫切的问题布局工作，努力扩大投资和消费需求，加快重点领域和关键环节改革，坚定发展信心，抢抓发展机遇，知难而进，逆势而上，实现经济平稳快速增长，为长沙率先发展增后劲，为富民强省做贡献，为人民群众谋福祉，推进创业之都、宜居城市、幸福家园建设。

今年全市经济社会发展的预期目标是：地区生产总值增长12%；地方财政收入增长13%；全社会固定资产投资增长25%以上；社会消费品零售总额增长16%；单位地区生产总值综合能耗下降4%，主要污染物排放总量减少4%；城乡居民人均可支配收入分别增长7%、9%；城镇登记失业率控制在5%以内；人口自然增长率控制在5.5％。以内；居民消费价格指数在省会城市平均水平以下。

落实今年工作要求，完成预定目标任务，必须认真贯彻落实中央和省、市委经济工作会议精神，把保持经济平稳快速增长作为经济工作的首要任务，把扩大投资、拉动消费作为保持经济平稳快速增长的根本途径，突出抓好重大项目建设，努力激活居民消费需求。按照有利于产业升级、有利于长远发展、有利于改善民生、有利于生态环保的原则，优先实施拉动作用大，群众受益大、受益快的项目，加快实施安居工程、畅通工程、品质工程、文化工程、民生工程、重点片区工程、生态节能工程、交通能源工程、农业水利工程和产业发展项目，突出抓好十大基础设施工程、十大民生社会工程、十大产业建设工程，力争完成重大项目投资1425亿元，把扩大投资作为拉动经济增长的最有效手段。

各位代表！尽管我们面临着巨大的压力和空前的挑战，但长沙发展的基本面没有变，多年形成的基础优势和内在动力没有变，加快发展的强烈愿望没有变。我们既要树立危机意识，更要树立机遇意识，善于从变化的形势中捕捉机遇，在逆境中培育有利因素，变压力为动力，迅速行动起来，上一批事关长远发展、事关民生保障的重大项目，干成多年想干而没有干成的大事，保持经济社会平稳快速发展。

三、2009年工作重点

实现2009年发展目标，要重点抓好以下几个方面的工作：

（一）突出重大项目建设，保持有效投资增长。项目是投资的载体，扩大投资是促进经济增长的直接动力。抓好重点项目建设。继续推进以“一江两岸”为重点的城建重点工程，续建湘江大道、潇湘大道北延线，完善橘子洲风景区建设，推进南湖、新河三角洲等片区建设。着力推进黄花机场改扩建、武广长沙站场及配套工程、长株潭城际轨道交通、京珠高速复线、石长铁路复线和中烟物流等中央、省在长重大基础设施和产业项目。加强项目招商引资。发挥协会、商会和中介机构的作用，实行以商招商、定向上门招商，办好招商节会。完善重大项目全程代办和绿色通道制度，加快项目落地。放宽经营性和准公益性领域准入门槛，引导社会资本投入。通过优质服务、优化环境，努力打造投资“洼地”，让财富向长沙汇聚。力争全年引进境外资金20亿美元，市外境内资金完成固定资产投资550亿元。创新项目投融资渠道。加强与中央和省的衔接，实行项目跟进，争取更多的项目投资。加快整合政府投资公司，打造片区建设融资平台。推进信用征信、信用担保体系建设，实施政府与金融机构的系统合作。开展城市建设集合委贷和资产证券化，支持企业上市融资。设立投资基金，引进风险投资、创业投资和券商、保险机构。

（二）突出产业升级，促进新型工业化。抓住结构调整的战略机遇，加快产业升级和经济转型。突出发展“两型产业”。推进企业技术改造、自主创新和兼并重组，支持企业自主研发，完成技改投入500亿元。着力推进技术标准化，加快科技成果产业化，办好科技成果转化交易会，推进知识产权示范市建设，力争科技成果转化率提高2个百分点，高新技术产业总产值增长20%以上。以长永高速、金洲大道为轴心,以高新区、经开区、生物医药基地为主体，打造“两型产业”聚集区。壮大优势产业集群。重点培育核心龙头企业，聚集发展多元配套企业，延伸龙头企业上下游产业链条。促进六大产业集群发展，形成1个产值过600亿元、3个产值过300亿元的产业集群。主动承接沿海先进制造业和现代服务业转移，力争获批全国加工贸易梯度转移重点承接地。优化整合园区资源。坚持突出特色、优势互补、错位发展，明确“两区九园”功能定位，优化园区布局，防止无序竞争，着力解决园区同质化发展问题。创新园区运作机制，优化服务流程。加大土地储备力度，完善园区基础设施配套，增强园区承载功能。新型工业化是长沙经济发展的第一推动力，要集中精力谋工业，努力实现工业经济的新跨越。

（三）突出城乡统筹，推进农村改革发展。要把提高农民收入、夯实农业基础作为扩大内需的重要内容。加强农村制度建设。稳定和完善农村基本经营制度，建立农村土地交易市场，以农民增收为目的，推进农村土地依法、自愿、有偿流转。推进集体林权制度改革。整合涉农资金，健全农业投入增长保障机制，探索农村建设发展投融资新模式。逐步扩大农业政策性保险的品种和范围。积极发展现代农业。围绕十大农业主导产业，支持发展30家农业产业化龙头企业，择优扶持“十大标志性工程”，农产品加工业产值突破400亿元。规划建设超级稻丰产示范工程、优质油茶产业基地等项目。实施农产品质量安全管理和市场准入制度，推行农业标准化生产，强化重大动物疫病防控。培育农民新型合作组织，开展农村服务业改革试点。提升现代农业设施装备水平，发展循环农业、生态农业，打造浏阳河生态农业走廊。加快农村基础设施建设。推进大中型病险水库除险加固、大型灌区配套建设和骨干山塘改造，加快防洪工程建设。启动新一轮农村电网提质改造。完成农村公路硬化1500公里。发展户用沼气池2.5万个。结合土地综合整理，抓好新农村示范村和示范片建设。加强小城镇基础设施配套，推动新型农村社区建设。努力增加农民收入。壮大县域经济，推动农村劳动力就地就近转移就业。实施返乡农民工就业帮扶工程，完成农民技能培训3.4万人，新增转移农村劳动力6万人。加大农村扶贫开发力度，帮助农民开拓农产品销售市场。扩大内需，最大潜力在农村；实现经济平稳快速发展，基础支撑在农业；保障和改善民生，重点难点在农民。要拿出真招实策，舍得真金白银，夯实农业基础，增加农民收入。

（四）突出城市建设管理，打造现代宜居城市。按照规划整齐、风格简洁、建筑精致、人与自然和谐的要求，推进现代宜居城市建设。继续实施品质工程。发挥规划龙头作用，启动新一轮城市总体规划修编；严格执行规划，提升规划水平。城市建设要更加注重集中连片，实施高密度、组团式开发，提高土地和基础设施利用效率，降低后续管理和配套建设成本。加强精细化管理，推行街道管理网格化、社区管理物业化。继续开展户外广告及违章建筑整治，加快实施景观街改造和站厕建设。加强宣传引导和制度约束，培育文明行为习惯，提升市民整体素质，巩固文明创建成果。着力解决居民住房问题。把安居工程作为宜居城市建设的首要任务，引导住房消费，消化存量房产，促进房产市场持续健康发展。推进“城中村”改造。采取新建和政府回购等方式，增加廉租房供给。加大棚户区改造力度，坚持政府主导，棚户区改造与经济适用房货币补贴相结合、与危旧房改造相结合，力争拆迁棚户区200万平方米。当我们的城市日新月异的时候，一定要让棚户区的居民共享城市发展成果。推进重大交通工程建设。继续实施畅通工程，修编综合交通规划。推进过江通道、城市主干道、片区路网建设，启动地铁、湘江航电枢纽工程建设，抓好堵点改造和市政道路大中修。加快长浏高速、G319宁乡段绕城线和霞凝港三期工程建设。实施公交优先战略，启动城市公共客运改革，加快客运、公交场站建设，加快公交汽车更新速度，着力提高公交品位和服务质量。建设食品安全城市。坚持惩防并举、城乡并重，健全食品安全监管机制，集中整治食品生产经营秩序，抓好种养、加工、流通、消费等环节监管，建立“从农田到餐桌”绿色供应体系，保障食品安全，用三至五年时间，将长沙基本建成全国食品安全城市。现代宜居城市是我们的共同追求，要通过不懈努力，使环境更加优美，生活更加方便，道路更加通畅，城市魅力更加凸显。

（五）突出综合配套改革，推进“两型社会”建设。探索资源节约、环境友好的发展道路，是历史赋予我们的重任。集中力量加快先导区建设。推进重点建设项目“三年行动计划”，加快枫林西路等12条道路、滨江新城等片区开发、英雄岭500KV变电站等配套设施建设，启动梅溪湖国际服务功能区、创新科技园和金融后援基地建设。加快推进产业发展。加强资源能源节约利用。大力发展资源节约型产业，建设节约型城市。严格执行产业和基础设施建设用地、能耗、环保等标准，突出抓好重点领域、重点企业节能和重点建筑节能节水改造。全面实施环保三年行动计划。突出农村面源污染治理，基本建成农村垃圾收集处理系统，完成浏阳、宁乡标准化生活垃圾无害化处理场建设。推进城市林业生态圈建设，抓好岳麓山景区提质改造。加快在建污水处理厂和城区纳污管网建设，加强湘江流域长沙段等饮用水源保护。巩固渣土、扬尘、噪音和机动车尾气污染治理成果，城市空气质量优良率保持90%以上，着力构建山青、水碧、天蓝的生态环境和秀丽景观。推进重点单项改革先行突破。加快城乡统筹、资源管理等综合配套改革，突出抓好土地管理改革，推行城乡建设用地增减挂钩、“先征后转”试点，推进土地节约集约利用，合理供应土地，提高利用效益。

（六）突出激活消费需求，拓展国内外市场。促进消费是扩大市场需求、保持经济增长的关键环节。加快现代服务业发展。优化商业网点规划布局，加强芙蓉中央商务区建设，努力培育新的城市商圈。发展会展经济，加大全国性会展申办力度，着力办好大型展会。大力发展文化教育、医疗保健和社区便民服务业。完善城乡市场体系，推进农村商品交易市场、商品配送中心、“万村千乡”市场工程建设。继续推进农贸市场提质改造。培育新的消费热点。大力发展消费性服务业，激活城乡居民消费潜能。拓展旅游消费，加快靖港古镇、灰汤温泉等旅游资源的保护性开发，扶持发展绿色生态旅游和“农家乐”休闲旅游，争取新设国际航线和旅游包机。落实家电下乡财政补贴政策，促进农村消费升级。拓展对外贸易。加强服务外包基地建设，积极申报国家工程机械出口基地、新的外派劳务基地和出口加工区，大力发展服务外包和加工贸易。完善金霞保税物流中心建设，加强“五定班列”和口岸综合管理，加快打通新的出口通道，促进口岸经济发展。力争进出口总额增长16%以上。通过扩大需求，有效吸收和消化企业产能，不断增强发展的协调性和持续能力。

（七）突出创业富民，建设创业之都。就业是民生之本，创业是发展之基，适宜各类人群就业创业是城市的最高追求。大力扶持创业就业。坚持人才是第一资源，实施人才强市战略。认真落实推进创业富民的有关政策，加快留学生创业园等创业基地建设。积极开展职业技能培训，建立城乡统筹的创业、就业扶持机制和服务体系，促进创业就业。帮扶困难企业和劳动密集型企业稳定就业岗位，实施公益性就业岗位援助，突出关心大学毕业生、返乡农民工和新增就业人员就业，新增城镇就业8万人，保持城镇零就业家庭动态清零。支持中小企业创业发展。落实好中小企业服务体系建设专项规划，完善中小企业担保体系，加大银行信贷和财政支持力度，发展中小企业集合债券，鼓励中小企业向专、新、特、精方向发展，增强市场竞争力，提高抗风险能力和就业吸纳能力。引导鼓励非公有制经济发展。优化非公有制经济发展环境，鼓励民间资本投入基础设施、国企改革、公用事业及其他非禁止性行业，支持非公有制企业进入国际市场，搭建非公有制企业与政府、银行、投资和担保机构合作平台，使非公有制经济总量不断壮大。努力使长沙成为要素集聚、成本较低、功能配套的区域性创业中心。

（八）突出改善民生，推进社会建设。改善民生是社会建设的重点，是落实科学发展观的核心内容。推进教育强市建设。要像抓重点工程一样抓教育，更多地关心教师、关心学生，促进教育公平和均衡发展。加强薄弱学校和农村学校建设，促进职业教育、高等教育、民办教育健康发展。开展危旧校舍的排查和加固，一定要把我们的学校建设成为最安全的地方，让学生安全、老师安心、家长放心。抓好文化工程建设。加强公共文化设施建设和公共文化服务，新建一批示范性乡镇（街道）综合文化站和基层文化阵地，深入推进农家书屋工程，加快广播电视村村通建设。加大历史文化街区（巷、古镇）、历史文化遗存和非物质文化遗产保护力度。进一步扶持文化支柱产业发展。建设健康城市。充分发挥社会体育设施的作用，加强公共体育设施建设，提高效用效率。广泛开展全民健身和爱国卫生运动，提升全民健康指数。组织和办好全国第十届中学生运动会、第七届长沙市运动会。启动公共卫生中心建设，加快村卫生室标准化建设，实现社区医疗卫生服务中心达标。认真抓好农村药品供应、监督网络建设。开展计划生育“三级联创四到基层”活动。健全城乡社会保障体系。抓好社会保险扩面工作，启动新型农村养老保险制度试点，健全城镇职工和居民基本医疗保障制度，提高“新农合”筹资水平和补偿标准。加强社会救助、社会福利、慈善和红十字会工作。提质改造乡镇敬老院10所，推进社区居家养老服务工作。改造农村特困户危房2000户。推进和谐长沙建设。越是困难时期，越要高度重视社会的安全平稳运行。夯实信访基础工作，热情诚恳地接待群众来信来访。加强仲裁、复议和人民调解工作，及时化解各类矛盾纠纷。推进和谐社区建设。加强统计工作，抓好第二次经济普查。发挥宗教界的积极作用，促进民族团结进步。加强国防教育、人民武装和国家安全工作。深入推进平安长沙建设，加强法制宣传教育、社会治安综合治理和公安基层基础建设。完善突发事件应急管理机制，突出抓好重点领域安全生产和专项整治，切实防止群体性事件和重特大安全生产事故发生。

（九）突出转变职能，建设人民满意政府。建设人民满意的政府是执政为民的要求，也是全市人民的期盼。深化行政管理体制改革。根据中央和省的统一部署，启动新一轮行政体制改革。全面推行阳光审批，审批时限再压缩30%以上。加快电子审批网络和电子监察系统建设，行政许可项目在线审批达70%。加强基层政权建设，开展创建“全国村务公开民主管理示范县（市、区）”活动。严格依法行政。规范行政程序，实行重大决策专家咨询论证、合法性审查、集体决定及后评价制度。规范执法行为，提高行政执法水平。自觉接受人大法律监督、工作监督和政协的民主监督，加强与民主党派、工商联的对口联系。发挥监察、审计等部门职能作用，加强媒体监督和社会监督，让人民更有效地监督政府。突出政风建设。政府是为人民服务的，政府干什么、怎么干，要广泛听取群众意见，充分尊重群众意愿，从群众最急需的事情做起，从群众身边的事情做起，继续抓好为民办实事工程。开展“帮助扶持企业发展，帮助推进项目建设，促进民生改善，促进经济增长”和“行政效能年”活动，深化行风评议，提高行政效率。强化公务员教育管理，完善绩效考核指标体系，推行公务员全员绩效考核，严格行政问责和行政过错责任追究，建设人民满意的公务员队伍。加强廉政建设。珍惜民力，艰苦奋斗，反对铺张，尽量降低行政成本，严格控制一般性支出，把有限的财力用到促进发展、改善民生上。严格要求，规范管理，廉洁自律，公开公平，努力建设节俭、清廉、对人民负责的政府。

各位代表，今年是新中国成立60周年。长沙正处在一个关键的时点上，挑战与机遇并存，只要我们万众一心，迎难而上，就一定能够战胜各种困难和挑战。让我们在市委的领导下，坚持科学发展、率先发展、和谐发展，加快创业之都、宜居城市、幸福家园建设，为率先基本实现全面小康而努力奋斗！
